# The detection of EpCAM^+^ and EpCAM^–^ circulating tumor cells

**DOI:** 10.1038/srep12270

**Published:** 2015-07-17

**Authors:** Sanne de Wit, Guus van Dalum, Aufried T. M. Lenferink, Arjan G. J. Tibbe, T. Jeroen N. Hiltermann, Harry J. M. Groen, Cees J. M. van Rijn, Leon W. M. M. Terstappen

**Affiliations:** 1 Department of Medical Cell BioPhysics, University of Twente, Hallenweg 23, 7522NH Enschede, the Netherlands; 2 VyCAP BV, Rademakerstraat 41, 7425PG Deventer, the Netherlands; 3 Department of Pulmonology, University Medical Center Groningen, University of Groningen, Hanzeplein 1, 9713GZ Groningen, the Netherlands; 4 Laboratory of Organic Chemistry, University of Wageningen, Dreijenplein 8, 6703HB Wageningen, the Netherlands

## Abstract

EpCAM expressing circulating tumor cells, detected by CellSearch, are predictive of short survival in several cancers and may serve as a liquid biopsy to guide therapy. Here we investigate the presence of EpCAM^+^ CTC detected by CellSearch and EpCAM^–^ CTC discarded by CellSearch, after EpCAM based enrichment. EpCAM^–^ CTC were identified by filtration and fluorescent labelling. This approach was validated using different cell lines spiked into blood and evaluated on blood samples of 27 metastatic lung cancer patients. The majority of spiked EpCAM^+^ cells could be detected with CellSearch, whereas most spiked cells with EpCAM^low^ or EpCAM^–^ expression were detected using filtration. Five or more CTC were detected in 15% of the patient samples, this increased to 41% when adding the CTC detected in the discarded blood. The number of patients with CTC and the number of CTC detected were doubled by the presence of EpCAM^–^ CTC. In this pilot study, the presence of EpCAM^+^ CTC was associated with poor outcome, whereas the EpCAM^–^ CTC were not. This observation will need to be confirmed in larger studies and molecular characterization needs to be conducted to elucidate differences between EpCAM^–^ and EpCAM^+^ CTC.

Circulating tumor cells (CTC) are cancer cells disseminated into the blood from primary or metastatic sites. The presence of CTC is predictive of relatively short survival in several types of cancer, including breast, prostate, colon, gastric, bladder, small and non-small cell lung carcinoma and melanoma^1^,^2^,^3^,^4^,^5^,^6^,^7^,^8^,^9^. At a concentration of 1 CTC in 1 mL of blood they are rare events, especially when compared to ~5·10^6^ white blood cells and ~5·10^9^ red blood cells per mL^10^,^11^. This implies that any assay for CTC enumeration must be able to handle the large number of normal cells. Selection of cells expressing the cell surface epithelial cell adhesion molecule (EpCAM) can be used for CTC enrichment as it has little or no expression on leukocytes and is expressed by the majority of epithelial derived cancers^12^,^13^,^14^. The FDA cleared CellSearch platform uses CTC enrichment by EpCAM targeted immunomagnetic selection, after which it identifies CTC among the enriched cells by expression of Cytokeratin’s 4–6, 8, 10, 13, 18 and 19, lack of CD45 expression, presence of a nucleus and cell like morphology^10^. CTC with this phenotype are associated with poor survival. An unresolved question is what the frequency and clinical relevance is of CTC, which do not have this phenotype and are thus currently not detected by the CellSearch platform. Here we present a method to investigate the presence of both EpCAM^+^ CTC and EpCAM^–^ CTC. This was achieved by the collection of the blood discarded by the CellSearch after immunomagnetic enrichment of EpCAM^+^ CTC, followed by enrichment of EpCAM^–^ CTC using filtration and immunofluorescent detection. In addition, CTC not expressing cytokeratin 4–6, 8, 10, 13, 18, or 19 were investigated by adding antibodies to cover all cytokeratins. This approach was validated using cells from tumor cell lines with different sizes and EpCAM densities. In a study of 27 metastatic lung cancer patients, we explored the presence of both the EpCAM^+^ CTC and EpCAM^–^ CTC.

## Results

### Capture efficiency of fluorescently labeled spiked cell lines

**Figure 1:**
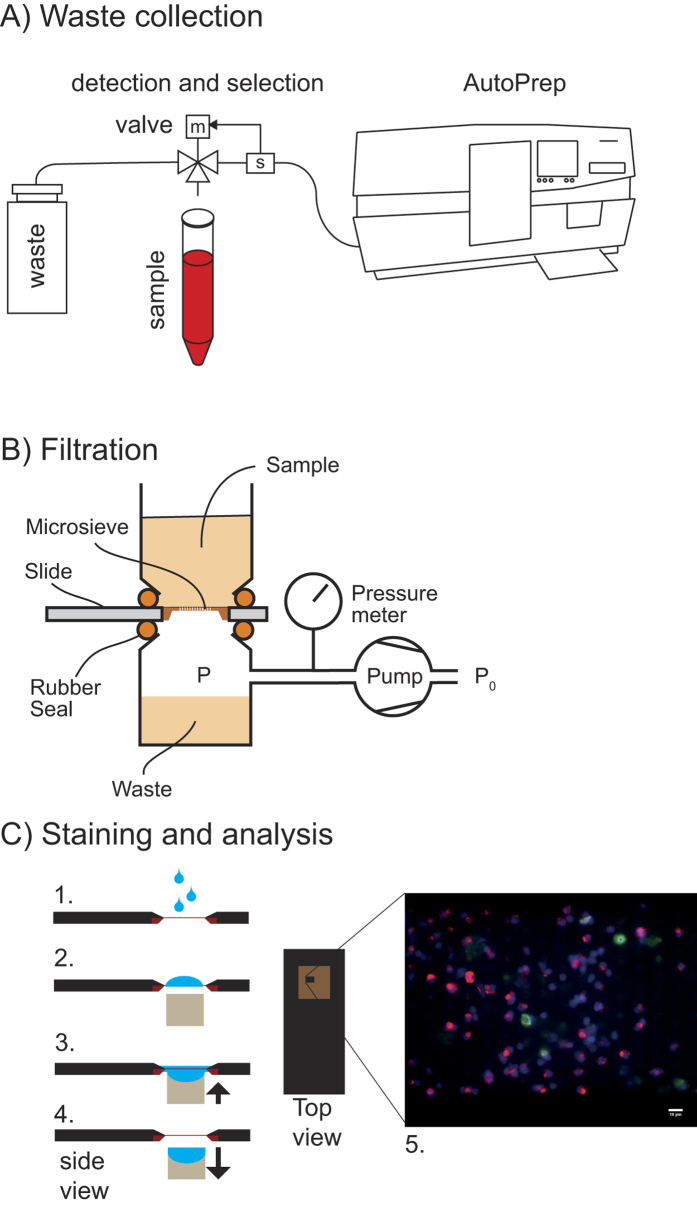
A schematic representation of the waste collection and filtration, followed by analysis of the microsieve with CTC. The Automatic Sample Collection Device collects the blood discarded after immunomagnetic enrichment of EpCAM^+^ cells by CellSearch Autoprep (**A**). A pump with disposable filtration unit containing a slide with a microsieve filters the discarded blood (**B**). The staining of the cells is performed directly on the filter (**C**) by adding a staining cocktail (1), incubating (2) and removing the liquid by bringing the sieve in contact with an absorbing body (3 and 4). The microsieve is analyzed using fluorescence microscopy for detection of CTC (5).

Two aliquots of 7.5 mL of peripheral blood from five healthy donors were spiked with approximately 500 pre-labeled cells from the tumor cell lines T24, SKBR3, Colo-320, SW480 and NCI-H1650. The EpCAM antigen density of cells of each cell line was determined by flow cytometry and varied from hundreds of molecules to millions. The size was determined by Coulter counter pipette and was 11–12 μm for smaller cells and 16 μm for larger cells. When each sample was run in the CellTracks Autoprep, the blood discarded by the system was collected and passed through the filtration device as illustrated in Fig. 1. The numbers of T24, SKBR3, Colo-320, SW480 and NCI-H1650 on the microsieves and inside the CellSearch cartridges were counted. The average number of cells counted and the standard deviation in the CellSearch cartridge and on the microsieves for each of the cell lines are provided in Table 1. The EpCAM^high^ cells show a high recovery of cells in the cartridge, whereas the EpCAM^low^ cells are mainly recovered on the microsieve. Because all samples travel through the same waste tubing, this could be a theoretical cause for carryover between collected samples. To determine this carryover on the CellTracks Autoprep, a blood sample of a healthy donor without tumor cells was placed after each sample spiked with T24 and SKBR3 cells and run through the complete protocol. The waste of these samples was also collected and filtered to determine the carryover between samples. The average percentage of the spiked cells found in six healthy donor samples was 0.3% (±0.3) for T24 cells and 1% (±0.3) for the SKBR3.

### Capture and staining efficiency of unlabeled spiked cell lines

To evaluate the staining efficiency of the captured cells, cells from the T24 bladder cancer cell line and NCI-H1650 lung cancer cell line were spiked in blood of healthy donors. The average number of spiked cells identified in the CellSearch cartridge and on the microsieve is shown in Table 1. This also shows the percentage of identified cells being significantly lower as compared to the pre-stained cells; 23% versus 59% for T24 and 27% versus 60% for NCI-H1650. To evaluate the background of CTC identified by CellSearch and on the microsieves in the CellSearch Waste, blood samples of eleven healthy controls were used and minimal CTC (on average less than 1) were detected in these unspiked samples, also shown in Table 1.

### Identification of CTC in blood from metastatic lung cancer patients by CellSearch with additional cytokeratin antibodies

To determine whether or not additional anti-cytokeratin antibodies increased the number of detected CTC, samples from 27 metastatic lung cancer patients were stained with the additional antibodies. The patient demographics are shown in Table 2. The samples were reanalyzed using cytokeratin FITC and DAPI as the primary threshold to generate images of CTC candidates for review in the CellTracks Analyzer II, instead of the traditional cytokeratin PE and DAPI as the primary threshold. By combining the two reviews of each sample, the CTC were divided into three groups: 1. CTC only positive for CK-PE in the original CellSearch test; 2. CTC only positive for CK-FITC; and 3. CTC positive for both CK-PE and CK-FITC. Of all the CTC that were detected, 50% were stained with the added CK-FITC markers and this additional CK was the only CK stain present in 15% of these CTC. The percentage of patient samples with ≥1 CTC in 7.5 mL of blood increased from 41% to 52%, when adding the number of CTC identified by the extra cytokeratins. A comparable increase was shown for a threshold of ≥3 CTC from 19% to 26% and for ≥5 CTC from 15% to 19%. The results of these analyses are summarized in Table 3 and the number of cells scored in each sample can be found in Supplementary Table S1.

### Identification of CTC in blood from metastatic lung cancer patients discarded by CellSearch

**Figure 2:**
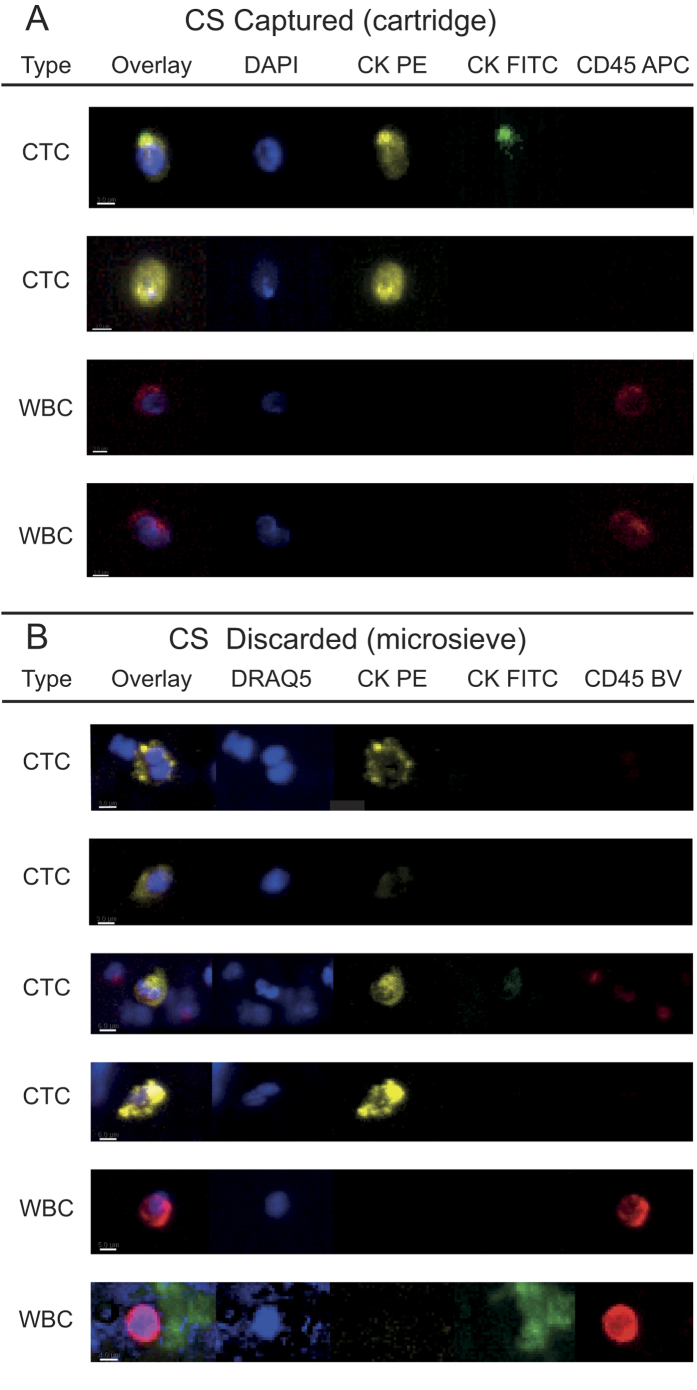
Thumbnail gallery of CTC and leukocytes in a lung cancer patient identified by CellSearch and in the blood discarded by CellSearch after filtration.

The number of CTC in the blood discarded by the CellTracks Autoprep was also analyzed in the samples from 27 metastatic lung cancer patients. The waste that belongs to a single blood sample of 7.5 mL, that is processed and then discarded by the Autoprep, has a volume of approximately 35 mL because of dilution with the system buffer during the CTC isolation process. Filtration of this waste volume of 35 mL takes 2–15 minutes. Figure 2a shows typical examples of images of CTC and leukocytes identified by CellSearch, whereas Fig. 2b displays typical images of CTC and leukocytes collected on the microsieve after filtration. The colors and scaling of the images was kept the same to obtain a fair comparison. When adding the number of CTC found in the waste by filtration, the percentage of patient samples with ≥1 CTC in 7.5 mL of blood increased from 41% to 74%. A similar increase was shown for a threshold of ≥3 CTC from 19% to 52%, for ≥5 CTC this increased from 15% to 41%, and for ≥10 CTC this increased from 11% to 26%. In the discarded blood, ≥1 CTC were detected in 33% of lung cancer patients in which no CTC were detected by CellSearch. This frequency decreases to 33%, 22% and 15% when thresholds were increased to ≥3, ≥5, ≥10 CTC respectively. CTC were found by CellSearch in 41% of the samples, while in 52% CTC were found in the waste using microsieve filtration. In 19% of the samples, CTC were detected by CellSearch and in the CellSearch Waste. There is no correlation between the number of both types of CTC in each sample with a Spearman’s Rho of 0.009. The number of cells found is summarized in Table 3 and can be found in more detail in Supplementary Table S1.

### CTC and overall survival

**Figure 3:**
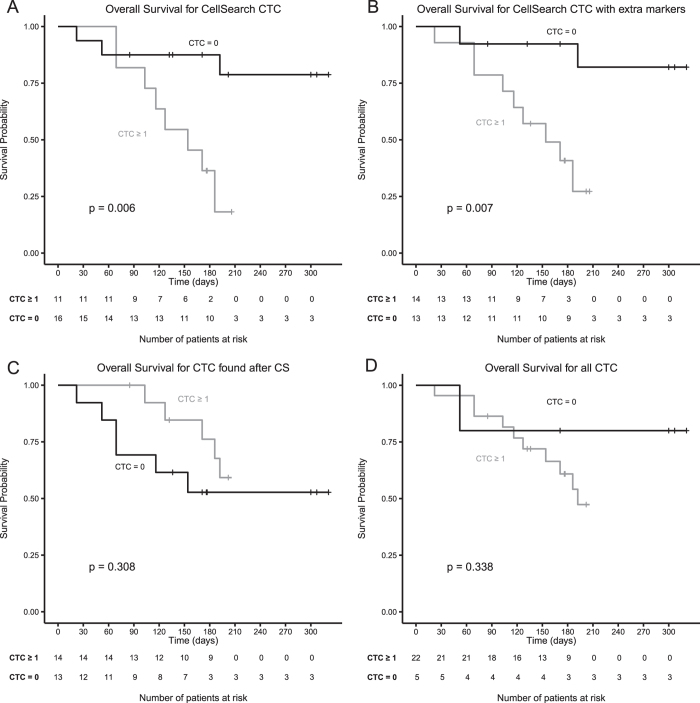
Kaplan-Meier curves for overall survival for CTC subpopulations with a cut-off of 1 CTC or more. Panel **A**; EpCAM^+^, CK 8,18^+^ or 19^+^ CTC detected by CellSearch. Panel **B**; EpCAM^+^, panCK^+^ CTC detected by CellSearch. Panel **C**; EpCAM^–^, panCK^+^ CTC after filtration of blood discarded by CellSearch (CS). Panel **D**; all populations of EpCAM^+^, panCK^+^ CTC and EpCAM^–^, panCK^+^ CTC.

To relate the presence of CTC to overall survival, the patient group was split into those with and those without detectable CTC. In Fig. 3 the Kaplan-Meier for the different CTC definitions are shown. Panel A shows a significant difference (p = 0.006) in overall survival when using CTC defined by CellSearch (41% patients with ≥1 CTC). Panel B also shows a significant difference (p = 0.007) in overall survival when using CTC defined by CellSearch with the addition cytokeratin coverage (52% patients with ≥1 CTC). Panel C shows no significant difference (p = 0.308) in overall survival when using the EpCAM^–^ CTC detected in the blood discarded by CellSearch (74% patients with ≥1 CTC). Panel D also shows no significant difference (p = 0.338) in overall survival when all CTC detected were evaluated (81% patients with ≥1 CTC). These results change slightly when the threshold was set at ≥5 CTC (see Supplementary Fig. S1 and Table 3). For CellSearch 15% of patients had ≥5 CTC with a significant difference in overall survival (p = 0.007). The significant association between CTC with additional cytokeratin coverage and overall survival is dependent on the threshold, 19% of patients had ≥5 CTC, but there was no longer a significant difference in overall survival (p = 0.075). For EpCAM^–^ CTC, 26% of patients had ≥5 CTC and no significant difference in overall survival was found (p = 0.526). Finally, for all CTC detected, 41% of patients had ≥5 CTC and no significant difference was noted (p = 0.118).

## Discussion

Treatment options for patients with metastatic cancer are increasing and create a need for biomarkers to determine whether the tumor will respond to the intended therapy. To enable quantitative real-time drug responsiveness, biomarkers need to be determined at multiple time points. The ability to obtain tumor cells from the blood would enable the ability to assess the presence of treatment targets on tumor cells real time. Prerequisite to achieve this goal is the presence of CTC in blood, which can be isolated, their identity verified and their composition determined. The heterogeneity of CTC within individual patients imposes the need for analysis of multiple CTC. Ten or more CTC are detected in 7.5 mL of blood in 11% of metastatic colorectal, 32% of metastatic breast and 40% of metastatic prostate cancer cases, using the CellSearch system, which is the current standard for CTC enumeration^11^. Although processing larger blood volumes could circumvent this issue^11^,^15^,^16^, the presence of CTC with a different phenotype, as those identified with the CellSearch system, may also increase the number of CTC detected. Requirement is that these CTC are also representative of the tumor and their presence is associated with poor outcome. Many alternative technologies have been introduced and compared with the results of the CellSearch system^17^,^18^,^19^,^20^,^21^,^22^,^23^,^24^,^25^,^26^,^27^. Most of these studies show the presence of more CTC, but do not address the question whether these identified cells have clinical relevance or the difference between the detected cells and the CellSearch CTC. To address the question which “CTC” are not detected by the CellSearch approach, we expanded the coverage of the cytokeratins used in the CellSearch system and we captured the blood discarded by the CellSearch system after immunomagnetic depletion of the EpCAM expressing cells in the blood and investigated the presence of CTC after filtration.

Cell lines with different sizes, EpCAM and cytokeratin expression were spiked in blood to determine the proportion of each cell type that can be detected. The capture efficiency of fluorescently pre-labeled spiked cell lines shows the expected results; recovery of the cells with CellSearch is proportional to the density of the EpCAM antigen and recovery of the cells on the microsieve after filtration is related to the size of the cells^28^. The experiments confirm that the CellSearch system is very efficient in recovery of cells with relatively high EpCAM expression. Low or no EpCAM expression significantly decreases the efficiency and a large portion of these cells can be captured in the blood discarded by the CellSearch system using filtration. Filtration efficiency, however, depends on the size of the cells and smaller tumor cells will still be missed using this approach. The configuration of the microsieves used for filtration was previously optimized for the filtration of whole blood^28^,^29^. The approximately 5-fold increase in volume by the dilution of the blood discarded by the CellSearch Autoprep does not seem to have a negative effect on the enrichment of cell lines by filtration, since the capture efficiency of cells from different cell lines was similar in whole blood^29^.

To investigate the influence of the staining protocol and microscopic examination on cell identification, unlabeled cells of different sizes were spiked in blood. Compared to the pre-stained cells, the percentage of identified cells was significantly lower (see Table 1). This observation demonstrates that improvements in CTC detection can be obtained by improving the staining and detection of these cells on microsieves.

Carryover between collected samples in the CellSearch Autoprep could theoretically occur, since all samples travel through the same waste tubing. Spiking experiments showed only a carryover of 1% and less, which is approximately one magnitude larger than with the CellSearch test, where carryover is possible when samples have a CTC count higher than 5000 cells. A maximum of 30 CTC were found in the CellSearch Waste of patient samples, therefore we do not expect carryover to have influenced our results. Control samples containing higher number of spiked cell lines were always placed at later stations in the CellSearch Autoprep when processed with patient samples in the same run, to make sure that patient samples would be collected first.

The percentage of patient samples with ≥1 CTC in 7.5 mL of blood increased from 41% to 81%, when adding the number of CTC found in the waste by filtration and adding the CTC identified by the extra cytokeratins. A similar increase was shown for a threshold of ≥3, ≥5 and ≥10 CTC. This increase in detected CTC number can be contributed mainly to the presence of CTC in the blood discarded by the CellSearch system, as can be seen in Table 3. It is likely these CTC express very little or no EpCAM. This was demonstrated by the processing of blood of healthy donors spiked with cell lines, and are therefore not detected by the CellSearch system.

The urging question is whether this increase in the number of CTC is also reflected in the clinical outcome of the patients. To start addressing this question, we asked the treating physicians after the study was completed for the last date of contact with the patients and the date of death, if that had occurred. The relation between overall survival and the type of CTC detected was determined and displayed using Kaplan-Meier curves, as shown in Fig. 3. We confirmed the relation between EpCAM^+^, CK 8,18^+^ or 19^+^ CTC detected by CellSearch and poor outcome (Fig. 3A). This relation was maintained by increasing the coverage of the cytokeratins, which resulted in more patients having CTC detected in blood (Fig. 3B). The level of significance depended on the CTC threshold and clearly shows the limitations of the small patient cohort, which were also diverse with respect to cancer type, line and type of therapy (see Table 2 and Supplementary Table S1). We did not find a significant relation between the presence of EpCAM^–^, panCK^+^ CTC and overall survival (Fig. 3C) and also no relation between overall survival and all classes of CTC detected (Fig. 3D). The number of patients is too small in numbers to make any definitive conclusions. Still, this pilot study warrants a larger study to confirm the findings. It also urges for an in-depth characterization of the EpCAM^+^ and EpCAM^–^ CTC to confirm by molecular analysis that the EpCAM^–^, panCK^+^ cells are indeed cancerous cells, as well as identifying antigens that may explain the difference in clinical behavior, for example the means for CTC to extravasate and form distant metastasis.

## Materials and Methods

### Lung cancer patients and healthy donors

Peripheral blood samples were drawn by venipuncture into 10 mL CellSave Preservative Tubes (Janssen Diagnostics, Huntingdon Valley, PA, USA) from healthy donors and metastatic lung cancer patients treated at the University Medical Center Groningen. Patient demographics are provided in Table 2. The experiments where done in accordance with relevant guidelines. All patients provided written informed consent and the study protocol was approved by the medical ethical committee of the University Medical Center Groningen (Groningen, The Netherlands). Healthy volunteers aged 20–55 gave written informed consent before donating blood.

### CTC Detection by CellSearch

CTC were enumerated in aliquots of 7.5 mL of blood with the CellSearch system (Janssen Diagnostics). Analysis was performed within 96 hours of the blood draw. Antibodies directed against the epithelial cell adhesion antigen (EpCAM) coupled to ferrofluids were used to enrich CTC. The enriched cells were fluorescently labeled with: the nucleic acid dye 4′6-diaminodino-2-phenylindole (DAPI), phycoerythrin (PE) labeled anti-cytokeratin monoclonal antibodies (mAbs) C11 and A53.B/A2 and Allophycocyan (APC) labeled mAb directed against CD45 (clone HI30) recognizing leukocytes using the CellSearch CTC kit (Janssen Diagnostics, Huntingdon Valley, PA, USA). To cover all cytokeratins, the Fluorescein (FITC) labeled anti-cytokeratin mAbs LP5K (Millipore, Billerica MA, USA) and Ks20.10 (Acris Antibodies, Herford, Germany) were added at 11.2 μg/mL and AE1/AE3 (eBioscience, San Diego CA, USA) was added at 5.6 μg/mL to the extra marker position in the CellSearch Epithelial Cell kit. The extra marker channel was used for the measurement of FITC on the CellTracks Analyzer II. Images of CTC candidates were identified by the CellTracks Analyzer II analyzing the sample twice, using either PE or FITC as the cytokeratin marker and presented to experienced operators for classification. Candidates were assigned as CTC when the objects were larger than 4 μm, stained with DAPI and cytokeratin, lacked CD45 staining and had morphological features consistent with that of a cell^10^.

### Blood waste collection of CellTracks Autoprep

After immunomagnetic selection of EpCAM^+^ cells, the CellTracks Autoprep aspirates the blood that is void of the selected cells and transports it to a waste container outside the instrument. To enable the investigation of this blood for residual tumor cells, a device was designed and build to collect this blood waste of the blood samples placed on the CellTracks Autoprep. The device was inserted between the waste tube from the CellTracks Autoprep system and the waste container. The device uses a LED and a photo diode to sense the presence of approaching blood in the waste tube and diverted the samples into standard 50 mL conical sample tubes using an actuated 3-way valve. A motorized rotor holding twelve 50 mL tubes was used to position the correct tube under the valve at the appropriate time. The waste collection device was controlled using a LabView (National Instruments, Austin TX, USA) program on a laptop computer. Figure 1a shows a schematic representation of the waste collection.

### Filtration of CellTracks Autoprep blood waste

The system to filter tumor cells from whole blood or the Autoprep waste comprises of a pump unit and a filtration unit holding a slide with a microsieve (VyCAP, Deventer, The Netherlands). The pump unit maintained a pressure of 100 mbar across the microsieve during filtration of the blood. A schematic image of this system is presented in Fig. 1b. The microsieve filtration membrane has a thickness of 1 μm supported by 350 μm thick Si and are atomically flat. The total surface area is 8 × 8 mm^2^ and contains 111,800 pores of 5 μm in diameter and are spaced 14 μm apart in lanes with a porosity of 10%. Specifications of the microsieves were obtained from previous experiments^28^,^29^. The CellSearch discarded sample waste was transferred to the filtration unit after which the pump was switched on. The collected sample waste of 35 mL was passed through in 2–15 minutes. This processing time is donor dependent. After completion of the filtration, the pump was switched off and the slide containing the microsieve is removed for staining.

### Staining of cells on microsieves

Conditions for staining on microsieves were optimized to assure uniform staining across the microsieve with a minimum of non-specific binding. After filtration the microsieve was removed and washed with PBS-saponin 0.15%. Next, a permeabilization buffer of PBS with 0.15% saponin (Sigma-Aldrich, St. Louis MO, USA) was placed on the sieve and removed after 15 minutes incubation at room temperature. A cocktail of fluorescently labeled antibodies was used to stain the cells on the sieve for 15 minutes at 37 °C using a heating plate (StatSpin, Westwood MA, USA). The staining solution consisted of 3 μM of the nucleic acid dye DRAQ5 (CellSignaling, Danvers MA, USA) and the following monoclonal antibodies: two antibodies targeting CK 4–6, 8, 10, 13, 18 & 19 (C11 and A.53B/A2, Janssen Diagnostics) labeled with PE, three antibodies targeting CK 1–8,10,14–16, 19 &20 (AE1/AE3, LP5K and Ks20.10) labeled to FITC and one antibody targeting CD45 (HI30) labeled with Brilliant Violet 421 (Biolegend, San Diego CA, USA). All antibodies were diluted to a final concentration of 1 μg/mL (HI30, C11, A.53B/A2 and AE1/AE3) or 2 μg/mL (LP5K and Ks20.10) in PBS containing 1% bovine serum albumin (Sigma) and 0.05% saponin. After removal of the staining cocktail, the microsieve was washed 2 times with PBS-BSA 1%. Then the sample was fixed using PBS with 1% formaldehyde (Sigma) for 10 minutes at room temperature. Removal of the fluid during each of the staining and washing steps was done by bringing the bottom of the microsieve in contact with an absorbing material using a staining holder (VyCAP), as illustrated in Fig. 1c. The microsieve was subsequently covered with PVA-DABCO (Sigma) mounting medium solution containing 3 μM of DRAQ5. A custom cut 0.85 × 0.85 cm^2^ glass coverslip (Menzel-Gläser, Saarbrükener, Germany) was placed on top of the microsieve for immediate analysis or storage in the freezer at −30 °C.

### Cell lines and spiking

Spiking experiments were performed with cells from the bladder carcinoma cell line T24, the breast carcinoma cell lines SKBR3, the colorectal cancer cell lines Colo-320 and SW480 and the lung cancer cell line NCI-H1650. All cell lines were obtained from ATCC (Manassa, VA, USA) and have not been authenticated in the past six months. They were grown at 37 °C and 5% CO^2^. SKBR3 and SW480 were cultured in Dulbecco’s modified eagle medium (Sigma), NCI-H1650 and Colo-320 were cultured in RPMI-1640 (Sigma) and T24 was cultured in DMEM-F12 (Sigma). Culture media were supplemented with 10% fetal calf serum (Gibco, Invitrogen, Carlsbad, CA, USA), 1% L-glutamin (Sigma) and 1% penicillin-streptomycin (Gibco). The median cell size was determined with a Coulter counter pipette (Scepter, Millipore, Billerica, MA, USA). The EpCAM density was determined using a flow cytometer (FACS ARIA II, BD Biosciences, San Jose, CA, USA) and QuantiBrite beads (BD Biosciences). For characterization of the microsieve performance, T24, Colo-320 and NCI-H1650 cells were stained with 5 μM CellTracker Orange CMTMR and SKBR-3 and SW480 cells with 25 μM CellTracker Green BODIPY (both Invitrogen, Carlsbad CA, USA). Cells were incubated in culture media for 24 hours at 37 **°**C with CellTracker prior to harvesting with 0.05% Trypsin-EDTA (Gibco). The prestained cells were spiked in healthy donor CellSave blood to determine filtration recovery. Cell numbers for spiking were counted with TruCOUNT Tubes (BD Biosciences) by flow cytometry. To determine the efficiency of the staining solution, spikes of approximately 300 NCI-H1650 cells were counted manually and subsequently added to healthy volunteer blood samples. The exact numbers of cells counted were used to determine the recovery on the sieve. Unspiked blood samples from healthy volunteers were used as a negative control.

### Detection of cells on microsieves

A Nikon E400 fluorescence microscope equipped with a Mercury Arc lamp as light source, a 10X (0.45NA) objective (Nikon, Tokyo, Japan), a computer-controlled CCD camera (Hamamatsu Photonics, Hamamatsu, Japan), X,Y,Z stage and 4-filter cube exchanger (LEP, Hawthorne NY, USA) with filters were used for acquisition of the fluorescent images covering the 0.64 cm^2^ surface of the microsieves. The following filters were used: APC with excitation 621/32 nm, dichroic 647 nm LP, emission 682/52 nm (Spectra Physics Newport, Santa Clara, CA, USA); BV421 with excitation 390/40 nm, dichroic 405 nm LP, emission 438/24 nm (Semrock, Rochester, NY, USA); FITC with excitation 480/20 nm, dichroic 495 nm LP, emission 510/20 nm (Spectra Physics Newport); and PE with excitation 547/12 nm, dichroic 560 nm LP, emission 579/25 nm (Spectra Physics Newport). The scanning and image acquisition was controlled by Labview (National Instruments, Austin TX, USA).

### Scoring of CTC

The CellSearch cartridges were analyzed twice, first to identify the traditional markers (DAPI+, CK-PE+, CK-FITC–, CD45–) and a dual positive CTC definition (DAPI+, CK-PE+, CK-FITC+, CD45–) after which the sample was reanalyzed for only the additional cytokeratin (DAPI+, CK-PE–, CK-FITC+, CD45–). A previously described image analysis algorithm^30^ written in MATLAB (MathWorks, Natick, MA, USA) was used to analyze the images taken from the microsieve and select likely CTC candidates by using a broader CTC classifier. The actual scoring of CTC was performed by an operator.

### Statistical analysis

Statistical analysis was done using R (R Foundation, Vienna, Austria ). A p-value less than 0.05 was considered to indicate a significant difference. Patients were divided into two prognostic groups: favorable for zero CTC and unfavorable for those with CTC. Kaplan-Meier curves for overall survival were generated and compared using the Log-Rank test.

## Additional Information

**How to cite this article**: de Wit, S. *et al.* The detection of EpCAM^+^ and EpCAM^–^ circulating tumor cells. *Sci. Rep.* **5**, 12270; doi: 10.1038/srep12270 (2015).

## Supplementary Material

Supplementary Data

## Figures and Tables

**Table 1:** Recovery of cell lines in healthy donor blood.

	Relatively large		Relatively small			
	T24	SKBR3	Colo-320	SW480	NCI-H1650	Healthy Control (N = 11)
	EpCAM^low^	EpCAM^high^	EpCAM^low^	EpCAM^high^	EpCAM^neg^	
EpCAM molecules	4.9 × 10^3^	1.5 × 10^6^	2.0 × 10^3^	2.3 × 10^6^	1.4 × 10^2^	
Size	16 μm	16 μm	11 μm	11 μm	12 μm	
Average recovery of pre-stained cells ± standard deviation (N = 5)						
CS recovery	2% (±1)	87% (±12)	2% (±2)	91% (±13)	0.2% (±0.3)	nd
MS recovery	59% (±9)	2% (±1)	18% (±6)	6% (±7)	60% (±7)	nd
Average recovery of cells not pre-stained ± standard deviation (N = 4)						
CS recovery	15% (±5)	nd	nd	nd	0.1% (±0.1)	0.3 (±0.9)
MS recovery	23% (±7)	nd	nd	nd	32% (±7)	0.3 (±0.6)

Recovery of cell lines spiked in blood of healthy donors and processed by CellSearch (CS) and the blood discarded by CS was collected and filtered through a microsieve (MS). The cells in the CS cartridges were counted on the CellTracks Analyzer II and the cells on the MS by standard fluorescent microscope.

**Table 2:** Patient demographics (N = 27).

Age (years)	
Average	62
Min-Max	32–82
Sex	
Male	52%
Female	48%
Type	
Adenocarcinoma	18
Squamous Cell carcinoma	4
Small Cell Carcinoma	3
Large Cell Carcinoma	2
Line of therapy	
1^st^ line	74%
≥2 line	26%
Status at last follow-up	
Alive	59%
Dead	41%
Average follow-up time in days (min-max)	
Alive	201 (85–321)
Dead	115 (22–192)

**Table 3:** Overview of patients (N = 27) with detected number of CTC.

Method	% Patients with CTC			
	≥1	≥3	≥5	≥10
CellSearch CTC	41%	19%	15%	11%
CellSearch CTC & additional CK marker CTC	52%	26%	19%	11%
CellSearch CTC & filtered CTC from CellSearch Waste	74%	52%	41%	26%
All types of CTC	81%	56%	41%	26%

Overview of CTC that are detected in 27 lung cancer patients. For detection with CellSearch, extra CTC with additional cytokeratin are separately included. CTC detected on the microsieves after filtration of the discarded blood from CellSearch are separately included as well. The final percentage of patients show the detected CTC from all above categories in total.
