# Supplementary material for: The detection of EpCAM+ and EpCAM– circulating tumor cells
Source: Sci Rep. 2015 Jul 17;5:12270. doi: 10.1038/srep12270 (PMC4505332; doi:10.1038/srep12270)
Supplement: Supplementary Data [file srep12270-s1.doc]

**The detection of EpCAM^+^ and EpCAM**^–^ **circulating tumor cells**

Sanne de Wit^#1^, Guus van Dalum^#1^, Aufried TM Lenferink^1^, Arjan GJ Tibbe^2^, T Jeroen N Hiltermann^3^, Harry JM Groen^3^, Cees JM van Rijn^4^, Leon WMM Terstappen^1^*

^1^ Department of Medical Cell BioPhysics, University of Twente, Hallenweg 23, 7522NH Enschede, the Netherlands

^2^ VyCAP BV, Rademakerstraat 41, 7425PG Deventer, the Netherlands

^3^ Department of Pulmonology, University Medical Center Groningen, University of Groningen, Hanzeplein 1, 9713GZ Groningen, the Netherlands

^4^ Laboratory of Organic Chemistry, University of Wageningen, Dreijenplein 8, 6703HB Wageningen, the Netherlands

^#^ Both authors contributed equally

* Corresponding author

**Supplementary Data**

**Supplementary** Figure S1 – Overall survival for CTC subpopulations with a ≥5 CTC cut-off.

**Supplementary** Table S1 – Detailed overview of CTC found in lung cancer patients.

**Supplementary Data**

**Supplementary** Figure S1 – Overall survival for CTC subpopulations with a ≥5 CTC cut-off.

Kaplan-Meier curves for overall survival for CTC subpopulations with a cut-off at 5 or more CTC. Panel A; EpCAM^+^, CK 8,18^+^ or 19^+^ CTC detected by CellSearch. Panel B; EpCAM^+^, panCK^+^ CTC detected by CellSearch. Panel C; EpCAM^–^, panCK^+^ CTC after filtration of blood discarded by CellSearch (CS). Panel D; all populations of EpCAM^+^, panCK^+^ CTC and EpCAM^–^, panCK^+^ CTC.


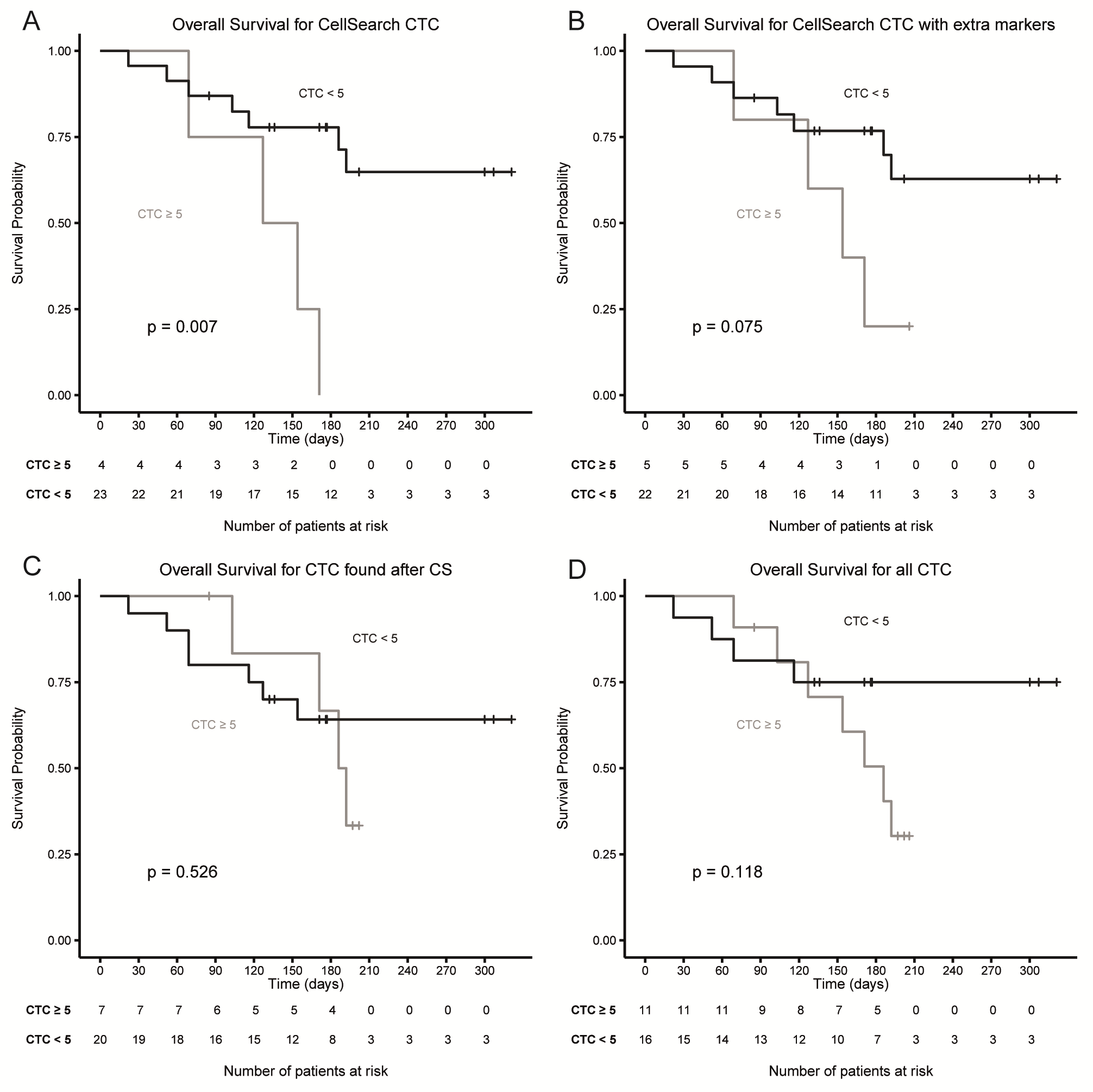


**Supplementary** Table S1 – Detailed overview of CTC found in lung cancer patients.

The left part shows the total number of CTC found using the traditional CellSearch count and the CTC found in waste filtration. The right part lists the CTC found using the FITC labeled CK cocktail. The events scored as CK FITC^+^ are the results of a reanalysis on the CellTracks of the same sample using the FITC channel for CK.

| **Patient #** | **CellSearch & EpCAM**^–^ **CTC** | | |  | **CellSearch extra cytokeratin markers** | | | | **Cancer**  type |
| --- | --- | --- | --- | --- | --- | --- | --- | --- | --- |
|  | **Total CTC** | **CTC**  **CellTracks** | **CTC Waste** |  | **CK PE+** | **CK PE+**  **CK FITC+** | **CK FITC+** | **Total** |  |
| **1** | 0 | 0 | 0 |  | 0 | 0 | 0 | 0 | Adeno |
| **2** | 0 | 0 | 0 |  | 0 | 0 | 0 | 0 | Squamous |
| **3** | 2 | 2 | 0 |  | 0 | 2 | 0 | 2 | Adeno |
| **4** | 0 | 0 | 0 |  | 0 | 0 | 2 | 2 | Squamous |
| **5** | 0 | 0 | 0 |  | 0 | 0 | 0 | 0 | Adeno |
| **6** | 11 | 11 | 0 |  | 2 | 9 | 0 | 11 | Adeno |
| **7** | 2 | 2 | 0 |  | 1 | 1 | 0 | 2 | Adeno |
| **8** | 31 | 1 | 30 |  | 1 | 0 | 1 | 2 | Squamous |
| **9** | 43 | 29 | 14 |  | 15 | 14 | 3 | 32 | Adeno |
| **10** | 16 | 2 | 14 |  | 2 | 0 | 0 | 2 | Adeno |
| **11** | 6 | 0 | 6 |  | 0 | 0 | 0 | 0 | Adeno |
| **12** | 1 | 0 | 1 |  | 0 | 0 | 0 | 0 | Adeno |
| **13** | 3 | 0 | 3 |  | 0 | 0 | 0 | 0 | Adeno |
| **14** | 6 | 2 | 4 |  | 2 | 1 | 2 | 5 | Large cell |
| **15** | 16 | 0 | 16 |  | 0 | 0 | 0 | 0 | Adeno |
| **16** | 2 | 0 | 2 |  | 0 | 0 | 0 | 0 | Adeno |
| **17** | 1 | 0 | 1 |  | 0 | 0 | 0 | 0 | Adeno |
| **18** | 5 | 0 | 5 |  | 0 | 0 | 1 | 1 | Small cell |
| **19** | 13 | 10 | 3 |  | 10 | 1 | 1 | 12 | Small cell |
| **20** | 0 | 0 | 0 |  | 0 | 0 | 0 | 0 | Adeno |
| **21** | 1 | 1 | 0 |  | 0 | 1 | 2 | 3 | Adeno |
| **22** | 4 | 4 | 0 |  | 4 | 0 | 0 | 4 | Adeno |
| **23** | 0 | 0 | 0 |  | 0 | 0 | 0 | 0 | Squamous |
| **24** | 6 | 6 | 0 |  | 6 | 1 | 0 | 6 | Small cell |
| **25** | 0 | 0 | 0 |  | 0 | 0 | 1 | 1 | Adeno |
| **26** | 3 | 0 | 3 |  | 0 | 0 | 0 | 0 | Adeno |
| **27** | 10 | 0 | 10 |  | 0 | 0 | 0 | 0 | Large cell |
